# BRAF^V600E^ Mutation and Its Association with Clinicopathological Features of Colorectal Cancer: A Systematic Review and Meta-Analysis

**DOI:** 10.1371/journal.pone.0090607

**Published:** 2014-03-03

**Authors:** Dong Chen, Jun-Fu Huang, Kai Liu, Li-Qun Zhang, Zhao Yang, Zheng-Ran Chuai, Yun-Xia Wang, Da-Chuan Shi, Qing Huang, Wei-Ling Fu

**Affiliations:** 1 Department of Laboratory Medicine, Southwest Hospital, Third Military Medical University, Chongqing, PR China; 2 Research Center for Nutrition and Food Safety, Institute of Military Preventive Medicine, Third Military Medical University, Chongqing, PR China; Yonsei University College of Medicine, Republic of Korea

## Abstract

**Background:**

Colorectal cancer (CRC) is a heterogeneous disease with multiple underlying causative genetic mutations. The B-type Raf proto-oncogene (BRAF) plays an important role in the mitogen-activated protein kinase (MAPK) signaling cascade during CRC. The presence of BRAF^V600E^ mutation can determine the response of a tumor to chemotherapy. However, the association between the BRAF^V600E^ mutation and the clinicopathological features of CRC remains controversial. We performed a systematic review and meta-analysis to estimate the effect of BRAF^V600E^ mutation on the clinicopathological characteristics of CRC.

**Methods:**

We identified studies that examined the effect of BRAF^V600E^ mutation on CRC within the PubMed, ISI Science Citation Index, and Embase databases. The effect of BRAF^V600E^ on outcome parameters was estimated by odds ratios (ORs) with 95% confidence intervals (CIs) for each study using a fixed effects or random effects model.

**Results:**

25 studies with a total of 11,955 CRC patients met inclusion criteria. The rate of BRAF^V600^ was 10.8% (1288/11955). The BRAF^V600E^ mutation in CRC was associated with advanced TNM stage, poor differentiation, mucinous histology, microsatellite instability (MSI), CpG island methylator phenotype (CIMP). This mutation was also associated with female gender, older age, proximal colon, and mutL homolog 1 (MLH1) methylation.

**Conclusions:**

This meta-analysis demonstrated that BRAF^V600E^ mutation was significantly correlated with adverse pathological features of CRC and distinct clinical characteristics. These data suggest that BRAF^V600E^ mutation could be used to supplement standard clinical and pathological staging for the better management of individual CRC patients, and could be considered as a poor prognostic marker for CRC.

## Introduction

Colorectal cancer (CRC) is the third most common cancer and the most frequent cause of cancer-related deaths worldwide, and so poses a serious threat to human health. [1], [2] It is widely accepted that CRC develops via a series of genetic and epigenetic changes that lead to the transformation of normal mucosa into a premalignant polyp, and ultimately to a malignancy. [3], [4] There are at least three different molecular pathways that lead to CRC. [5], [6] The chromosomal instability pathway is characterized by some of mutations such as P53 and v-Ki-ras2 Kirsten rat sarcoma viral oncogene homolog (KRAS). [7] The second is the mutator pathway, which involves the loss of function of DNA mismatch repair proteins secondary to germline mutations in mismatch repair genes. [8], [9] Finally, there is the serrated pathway. [10].

The B-type raf proto-oncogene (BRAF) encodes a serine/threonine kinase that plays a role in intracellular signaling and cell growth, and is a downstream effector of KRAS in the mitogen-activated protein kinase (MAPK) signaling pathway. [11], [12] The BRAF^V600E^ mutation, which accounts for approximately 90% BRAF mutations, is frequently observed in CRC with microsatellite instability (MSI). It arises from the serrated pathway, and occurs in 5–22% of patients. [11], [13] It was demonstrated that KRAS or BRAF^V600E^ mutations in CRC are associated with clinical resistance to treatment with epidermal growth factor receptor (EGFR)-targeted monoclonal antibodies. [14]–[16] However, the association between the BRAF^V600E^ mutation and the clinicopathological characteristics of CRC remains controversial. [11] Nevertheless, it would be valuable to supplement standard clinical and pathological staging using molecular markers such as KRAS and BRAF^V600E^ to more accurately classify subsets of patients for more effective clinical management. [13] Therefore, we aimed to estimate the effect of BRAF^V600E^ mutation on the clinicopathological characteristics of CRC.

We performed a systematic review and meta-analysis to quantify the association of the BRAF^V600E^ mutation with sociodemographic factors and clinicopathological characteristics of the CRC.

## Materials and Methods

### Eligibility Criteria for Meta-analysis

We searched extensively for studies that examined the association of BRAF^V600E^ mutation with clinicopathological characteristics. Our study had the following inclusion criteria: 1) BRAF^V600E^ mutation data from only CRC were included from articles that assessed clinicopathological characteristics. 2) Articles were published before July 2013 in English. 3) The newest or most appropriately informative single article was selected when the same authors or groups published multiple articles. 4) Relevant unpublished data that were presented at international meetings. The exclusion criteria were: 1) review articles without original data; 2) the absence of, or inappropriate clinicopathological data reported in the article; and 3) single case reports.

### Search Strategy

PubMed (http://www.ncbi.nlm.nih.gov/pubmed), ISI Science Citation Index (http://apps.isiknowledge.com), and EMBASE (http://www.embase.com/home) databases were searched using the keywords “BRAF”, “b-raf”, “colorectal”, “colon”, “rectal”, “rectum”, “cancer”, “neoplasm”, “tumor”, “malignant”, and “CRC” in different combinations, with the species being restricted to human. We also manually searched the reference lists of the articles identified in the searches for additional eligible studies. Duplications of data were carefully avoided by examining the names of all authors and the medical centers that participated in each publication. We contacted the authors for additional data when necessary.

### Data Extraction

The following information was extracted from each study: first author, publication year, country where the study was conducted, screening methods, number of patients, demographic features, clinicopathological characteristics, molecular features, lifestyle and frequency data including number of BRAF^V600E^ mutation in case group, total number of case group, number of BRAF^V600E^ mutation in control group, total number of control group. Two authors reviewed all studies independently, and disagreement was resolved by discussion with a third investigator. Two investigators crosschecked all data collected from the original articles, and each study was examined fully to eliminate duplicates.

### Quality Assessment

The quality of each study was assessed independently by 2 reviewers using the Newcastle-Ottawa Scale (NOS). [17] The NOS consists of 3 parameters for the quality of case-control study: selection, comparability, and outcome. The NOS assigns a maximum of 4 points for selection, 2 points for comparability, and 3 points for outcome. Studies with NOS scores >6 were consider high quality. [18] Any discrepancies between 2 reviewers were settled by a third reviewer.

### Primary and Secondary Outcomes

The primary outcome was advanced TNM stage, poor differentiation, mucinous histology, microsatellite instability (MSI), CpG island methylator phenotype (CIMP).Secondary outcomes were proximal colon, KRAS mutation, and mutL homolog 1 (MLH1) methylation and sociodemographic features of the patients including age, gender, smoking and alcohol intake.

### Data Pooling and Statistics

Meta-analysis was performed using RevMan (version 5) and Stata (version 11.0). Odds ratios (ORs) and the 95% confidence intervals (CIs) for each study were generated by inputting number of BRAF^V600E^ mutation in case group, total number of case group, number of BRAF^V600E^ mutation in control group and total number of control group into the RevMan. And the pooled effect size was defined as weighted OR with CI. Study heterogeneity was assessed using the chi-squared test of heterogeneity (Q Cochran’s Q statistic), and the Higgin’s I^2^ measure. Taking into account the low statistical power of these tests of heterogeneity, significant heterogeneity was defined as a Q test P value of <0.10, or an I^2^ measure >30%. ORs from the different studies were combined using fixed effects or random effects models. The choice of the fixed or random effects model was made on the absence or presence of significant heterogeneity based on the depended on the Q test, respectively. Sensitivity analysis was performed to assess the influence of each study on the pooled OR by serially omitting each individual study and pooling the remaining studies. Publication bias was assessed by visual inspection of the funnel plot for symmetry, and formal statistical testing using the Egger test.

## Results

**Figure 1:**
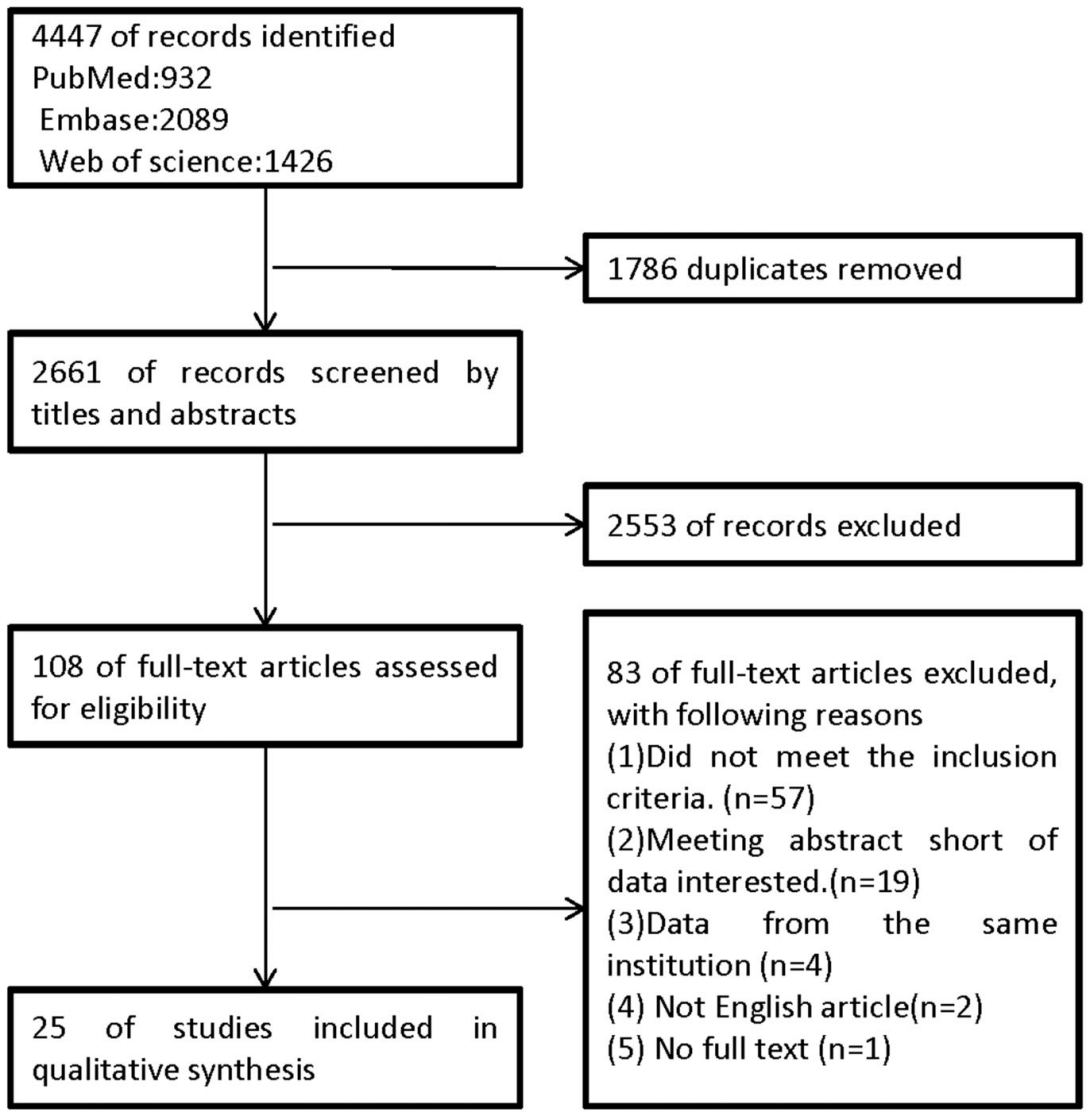
A flow chart highlighting study selection.

A total of 4447 abstracts and titles were obtained through electronic searches, and 1786 were excluded because of duplication. The titles and abstracts of the remaining 2661 records were then screened. An additional 2553 studies were excluded, and 108 full-text papers were deemed to be relevant and were examined in detail. Of these, 83 full-text articles were excluded for the reasons outlined in **Figure 1**. After these analyses, 25 studies with a total of 11,955 patients were included. Of the 11, 955 patients, 1288 had BRAF^V600E^ mutation-positive CRCs, giving an overall frequency of 10.8%. The earliest study was published in July 2005 by Samowitz et al. [19], and the most recent study was published in August 2012 by Phipps et al [13]. The largest study by Phipps et al. included 1980 patients [13], and the smallest study by Rako et al. included 71 patients [20]. Not all studies reported all variables examined in the meta-analysis, and so only studies that reported the variable of interest were analyzed for the association of BRAF^V600E^ with that variable. A summary of the 25 studies is listed in Table S1.

### BRAFV600E Mutation and Sociodemgraphic Characteristics of Patients with CRC

#### Gender

**Figure 2:**
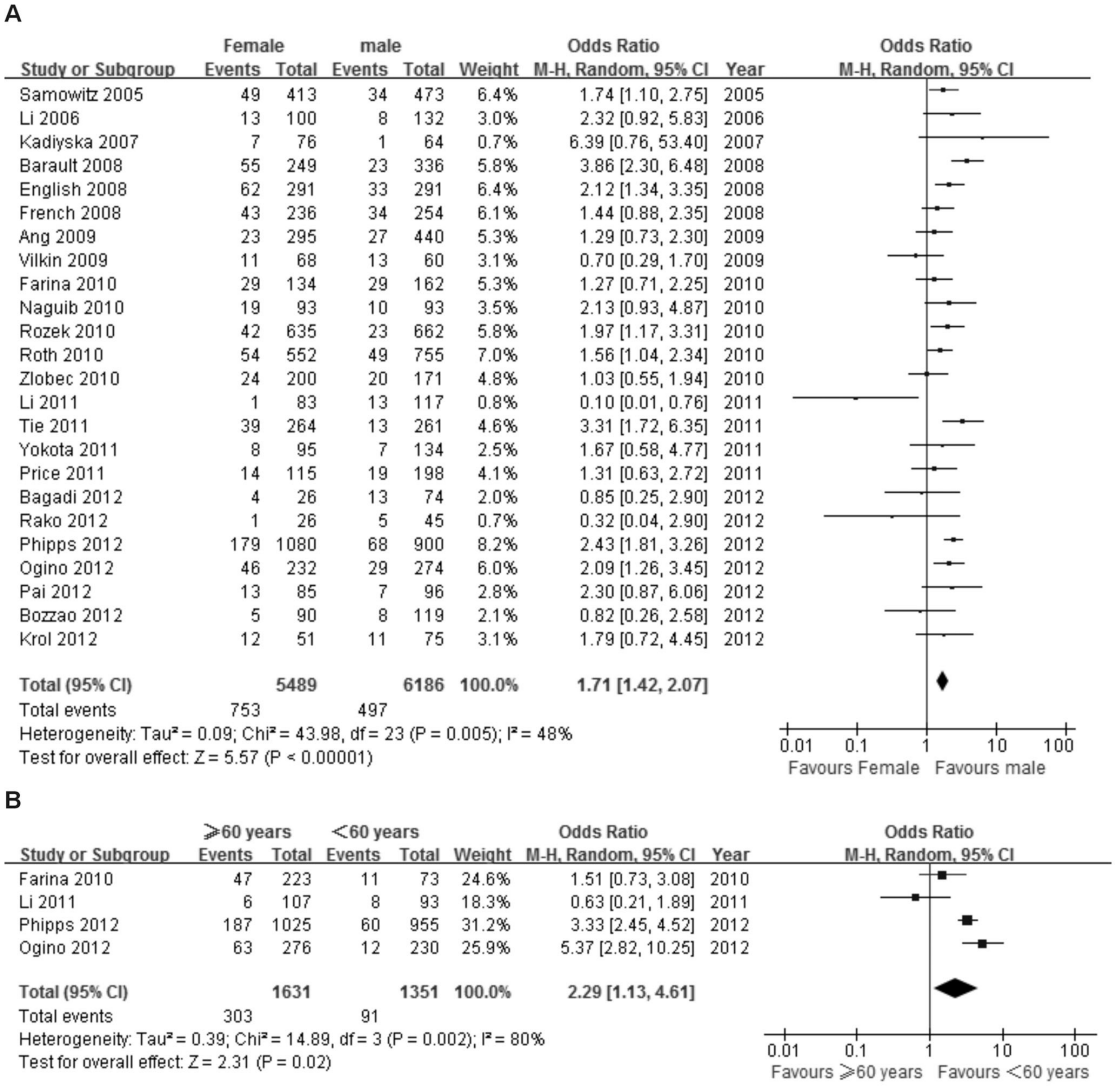
The association of BRAF^V600E^ mutation with demographics. Random effects model of the odds ratios (ORs) with 95% confidence intervals (CIs) for the association of BRAF^V600E^ mutation with gender (A) and age (B).

Twenty-four studies including 11,675 patients were analyzed for the association between BRAF^V600E^ mutation and gender. Of 5489 female patients, 753 (13.7%) were BRAF^V600E^ mutation positive, and 497 (8.0%) out of 6186 male patients were BRAF^V600E^ mutation positive. There was a significant association between BRAF^V600E^ mutation and female gender [OR = 1.71; 95% CI = 1.42–2.07] (Figure 2A).

#### Age

Four studies including 2982 patients were analyzed for the association between BRAF^V600E^ mutation and age. Of 1631 patients 60 years or older, 303 (18.6%) were BRAF^V600E^ mutation positive, compared with 91 (6.7%) of 1351 patients younger than 60 years old. The pooled analysis showed a significant association between BRAF^V600E^ mutation and age 60 years or older [OR = 2.29; 95% CI = 1.13–4.61] (Figure 2B).

#### Smoking

**Figure 3:**
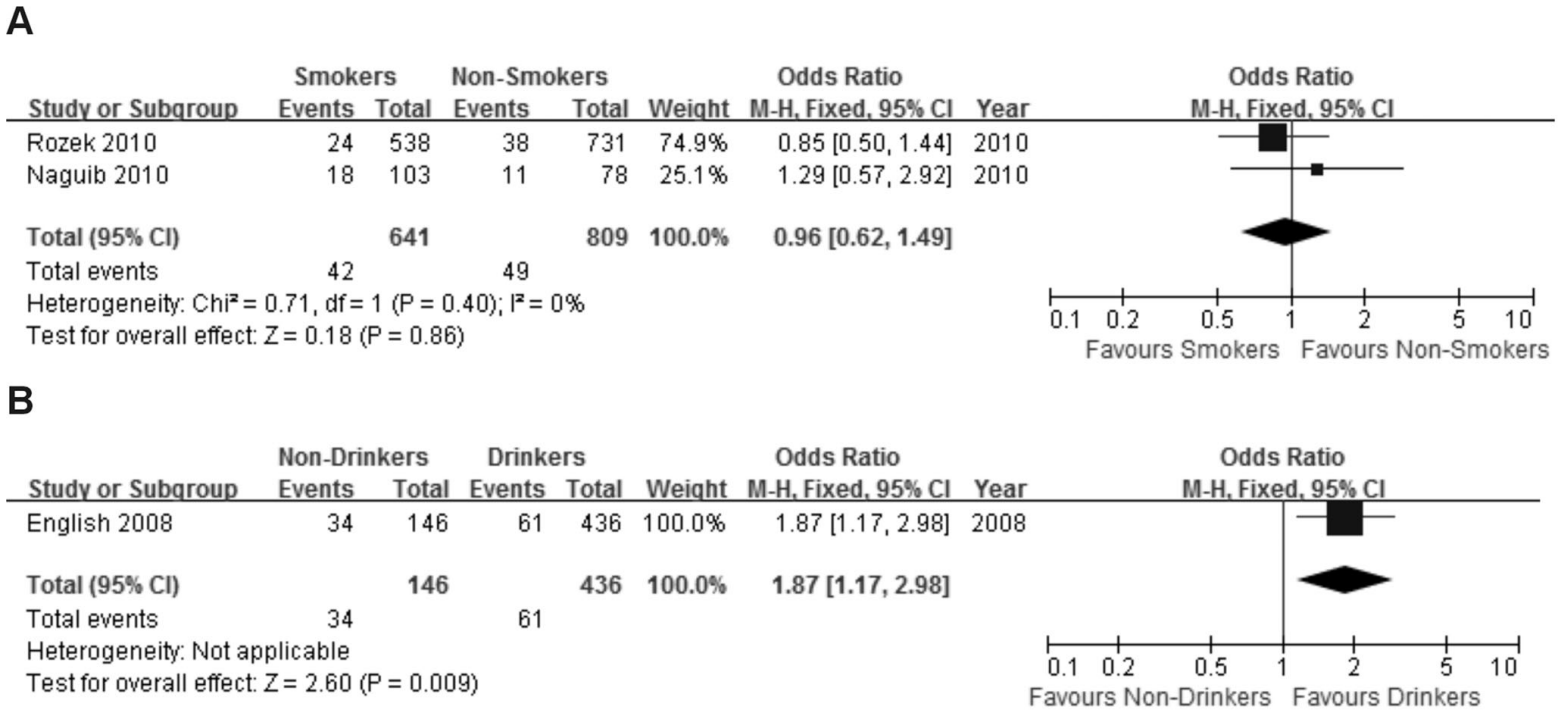
The association of BRAF^V600E^ mutation with life style. Fixed effects model of the odds ratios (ORs) with 95% confidence intervals (CIs) of the association of BRAF^V600E^ mutation with smoking (A) and alcohol consumption (B).

Two studies including 1450 patients were analyzed for the association between BRAF^V600E^ mutation and smoking. Of 641 smokers, 42 (6.6%) were BRAF^V600E^ mutation positive, compared with 49 (6.1%) out of 809 non-smokers. There was no significant association between BRAF^V600E^ mutation and smoking [OR = 0.96; 95% CI = 0.62–1.49] (Figure 3A).

#### Alcohol intake

Only one study including 582 patients analyzed the association between BRAF^V600E^ mutation and alcohol intake. Of 146 non-drinkers, 36 (24.7%) were BRAF^V600E^ mutation positive, compared with 61 (13.2%) out of 436 patients who drank alcohol. There was a significant negative correlation between BRAF^V600E^ mutation and alcohol intake [OR = 1.87; 95% CI = 1.17–2.98] (Figure 3B).

### BRAFV600E Mutation and Clinicopathologic Characteristics of Patients with CRC

#### TNM stage

**Figure 4:**
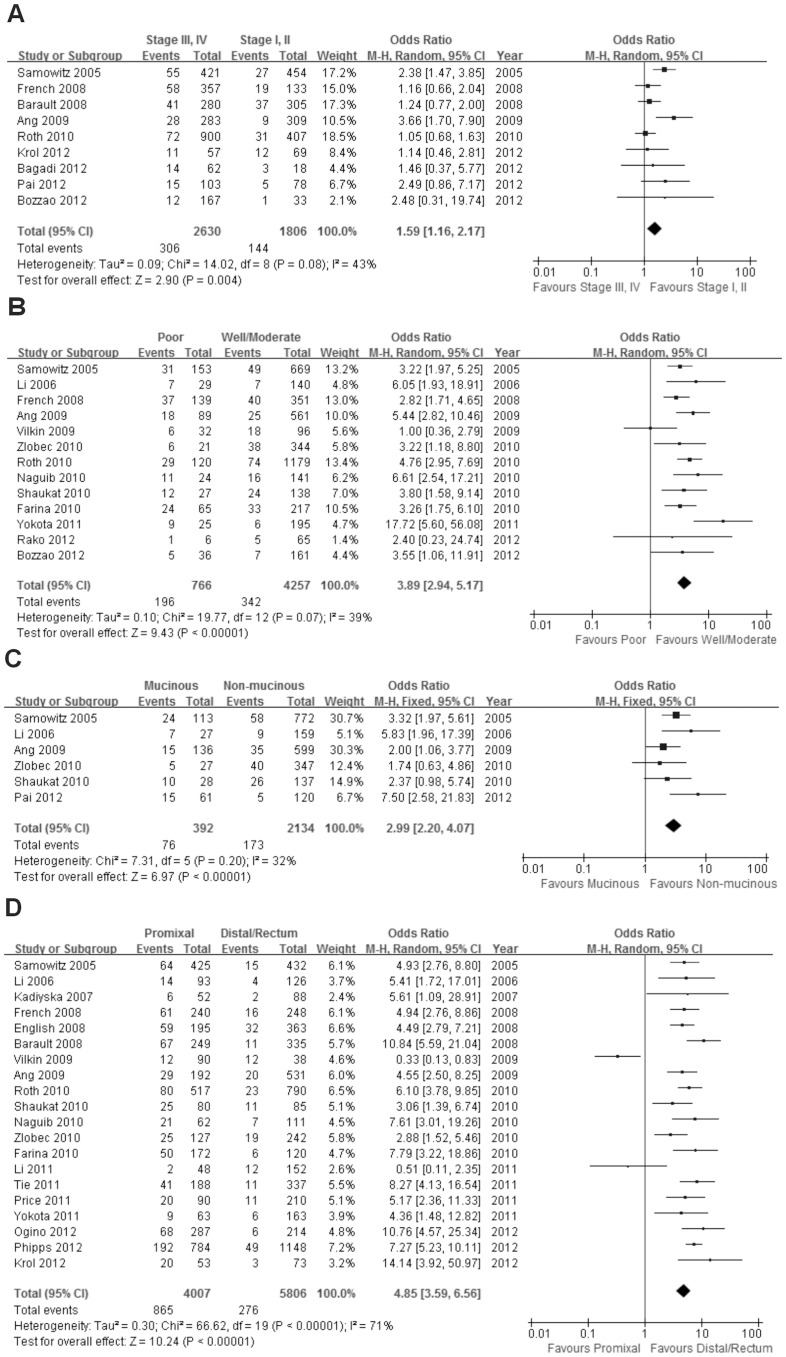
The association of BRAF^V600E^ mutation with clinicopathological features. Random effects model of the odds ratios (ORs) with 95% confidence intervals (CIs) of the association of BRAF^V600E^ mutation with clinical stage (A), tumor differentiation (B) and tumor location (D). Fixed effects model of the odds ratios (ORs) with 95% confidence intervals (CIs) of the association of BRAF^V600E^ mutation with mucinous histology (C).

Nine studies including 4436 patients were analyzed for the association between BRAF^V600E^ mutation and TNM stage (based on the AJCC classification) at diagnosis. Of 2630 patients with stage III or IV cancer, 306 (11.6%) were BRAF^V600E^ mutation positive compared with 144 (8.0%) of 1806 patients with stage I or II CRC. There was a significant association between BRAF^V600E^ mutation and advanced TNM stage at diagnosis [OR = 1.59; 95% CI = 1.16–2.17] (Figure 4A).

#### Differentiation

Thirteen studies including 5023 patients were analyzed for the association between BRAF^V600E^ mutation and colorectal differentiation. Of 766 patients with poor differentiation, 196 (25.6%) were BRAF^V600E^ mutation positive, and 342 (8.0%) of 4257 patients with well or moderately differentiated CRC were BRAF^V600E^ mutation positive. There was a significant association between BRAF^V600E^ mutation and poor differentiation [OR = 3.89; 95% CI = 2.94–5.17] (Figure 4B).

#### Mucinous histology

Six studies including 2526 patients were analyzed for the association between BRAF^V600E^ mutation and mucinous histology. Of 392 patients with mucinous histology, 76 (19.4%) were BRAF^V600E^ mutation positive, whereas 173 (8.1%) of 2134 patients with non-mucinous histology were BRAF^V600E^ mutation positive. There was a significant association between BRAF^V600E^ mutation and mucinous histology [OR = 2.99; 95% CI = 2.20–4.07] (Figure 4C).

#### Location

Twenty studies including 9813 patients were analyzed for the association between BRAF^V600E^ mutation and the location of the colorectal tumor. Of 4007 patients with tumors in the proximal colon, 865 (21.6%) were BRAF^V600E^ mutation positive, compared with 276 (4.8%) out of 5806 patients with distal colon or rectal tumors. There was a significant association between BRAF^V600E^ mutation and proximal colon tumor location [OR = 4.85; 95% CI = 3.59–6.56] (Figure 4D).

#### MSI status

**Figure 5:**
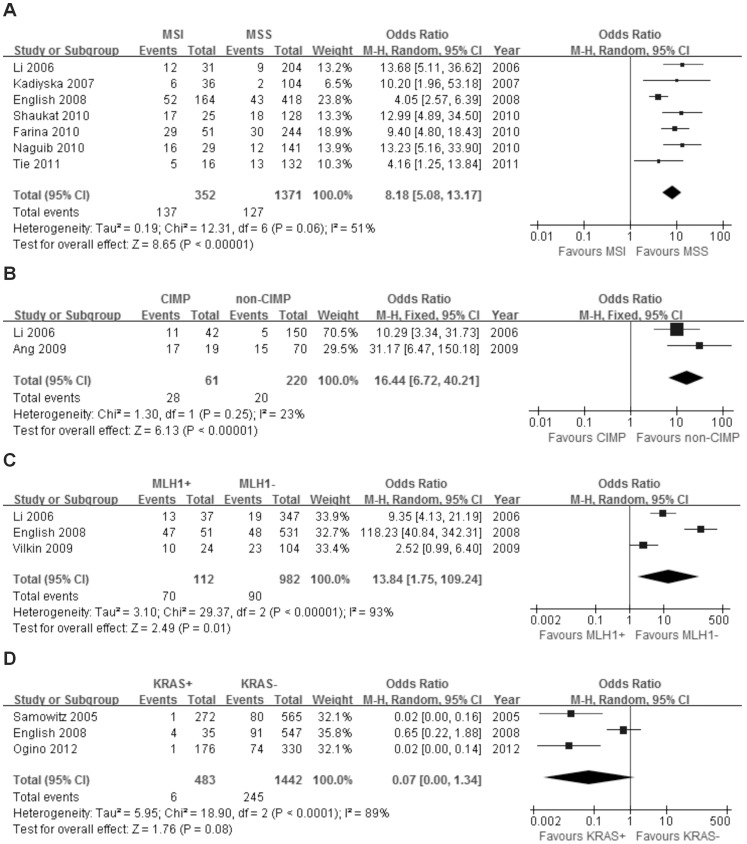
The association of BRAF^V600E^ mutation with molecular features. Random effects model of the odds ratios (ORs) with 95% confidence intervals (CIs) of the association of BRAF^V600E^ mutation with MSI status (A), MLH1 status (C) and KRAS mutation (D). Fixed effects model of the odds ratios (ORs) with 95% confidence intervals (CIs) of the association of BRAF^V600E^ mutation with CIMP status (B).

Seven studies including 1723 patients were analyzed for the association between BRAF^V600E^ mutation and MSI status. Of 352 patients with MSI, 137 (38.9%) were BRAF^V600E^ mutation positive, compared with 127 (9.3%) of 1371 patients with microsatellite stable (MSS) tumors. There was a significant association between BRAF^V600E^ mutation and MSI [OR = 8.18; 95% CI = 5.08–13.17] (Figure 5A).

#### CIMP status

Two studies including 281 patients were analyzed for the association between BRAF^V600E^ mutation and CIMP status. Of 61 patients with CIMP, 28 (45.9%) were BRAF^V600E^ mutation positive, compared with 20 (9.1%) out of 220 patients with non-CIMP tumors. There was a significant association between BRAF^V600E^ mutation and CIMP [OR = 16.44; 95% CI = 6.72–40.21] (Figure 5B).

#### MLH1 status

Three studies including 1094 patients were analyzed for the association between BRAF^V600E^ mutation and MLH1 methylation status. Of 112 patients with MLH1 methylation, 70 (62.5%) were BRAF^V600E^ mutation positive, whereas only 90 (9.2%) out of 982 patients with MLH1 non-methylated tumors were BRAF^V600E^ mutation positive. There was a significant association between BRAF^V600E^ mutation and MLH1 methylation [OR = 13.84; 95% CI = 1.75–109.24] (Figure 5C).

#### KRAS mutation

Three studies including 1925 patients were analyzed for the association between BRAF^V600E^ and KRAS mutation. Of 483 patients with KRAS mutations, six (1.2%) were BRAF^V600E^ mutation positive, compared with 245 (17.0%) out of 1442 patients without KRAS mutations. There was a significant association between BRAF^V600E^ mutation and wild-type KRAS [OR = 0.07; 95% CI = 0.00–1.34] (Figure 5D).

### Quality-Assessment

Twelve studies had an NOS score of 8, eleven studies had an NOS score of 7, 1 studies had an NOS score of 6, and 1 studies had an NOS score of 5. Twenty three studies (92%) were of high quality (NOS score >6), and the average NOS score was 7.36.

### Publication Bias and Sensitivity Analysis

To assess the presence of potential publication bias, a funnel plot was constructed depicting the effect sizes calculated from individual studies examining the association between BRAF^V600E^ mutation and the primary outcome. The funnel plot was symmetrical, suggesting the absence of significant biases. This was confirmed by results of Egger’s test (p = 0.332).

The sensitivity analyses revealed that no individual studies unduly influenced pooled ORs and CIs significantly, suggesting that the estimates were robust.

## Discussion

In the present study, we confirmed that the BRAF^V600E^ mutation in CRC was significantly associated with several clinicopathological factors. Within the studies included, the highest BRAF^V600E^ mutation rate was 21.8% in a study conducted in the United States reported by Shaukat et al. [21] The lowest mutation rate was 5.0% in a study completed in Israel by Rozek et al. [22] The BRAF^V600E^ mutation rate was significantly different between these two studies, which may be attributable to the different ethnicities of the study populations. The overall BRAF^V600E^ mutation frequency of 10.8% was similar to other reports in the literature. [19], [23], [24].

Our study, which contained a larger sample size, demonstrated that BRAF^V600E^ mutation was significantly associated with several sociodemographic and clinicopathologic characteristics in patients with CRC. The BRAF^V600E^ mutation was 1.71-fold more frequent in female patients with than males, whereas older patients were 2.29-fold more likely to carry the BRAF^V600E^ mutation than younger patients. The results obtained here suggest that the BRAF^V600E^ mutation is present more commonly in older and female patients, which is consistent with most previous studies. [22], [23], [25] Nevertheless, it was suggested by some studies that BRAF^V600E^ mutation was not associated with either female gender or older age. [24] This observation could be explained by the different sample sizes in the different studies.

This meta-analysis revealed that the BRAF^V600E^ mutation was significantly associated with advanced TNM stage, poor differentiation, mucinous histology, and tumors located in the proximal colon, which was consistent with previous reports. [21], [26]–[28] The results presented here alert physicians to patients that may be at increased risk of carrying a BRAF^V600E^ mutant tumor as the focus for screening. The gold standard prognostic factor for CRC is clinicopathological staging as well as other pathological factors, such as differentiation and histological subtype. [20].

In this meta-analysis the BRAF^V600E^ mutation was significantly associated with several the clinical and pathological factors. Therefore, we infer that BRAF^V600E^ mutations may play an important role in tumor development and the subsequent prognosis. To date, cancer has traditionally been classified based predominantly on microscopic morphology and immunophenotyping, but more rarely by molecular approaches. If the BRAF^V600E^ mutation, together with other molecular markers, could be used to supplement the current standard clinical and pathological staging for patients, it may improve overall patient care.

Our study revealed that the BRAF^V600E^ mutation was significantly associated with several molecular alterations. Tejpar et al. carried out a more detailed molecular analysis of CRCs to reveal that the molecular alterations in colorectal tumors can be heterogeneous. [29] Up to 85% of sporadic cases of CRC display chromosomal instability, which is characterized by mutations to genes such as TP53 and KRAS. The remaining 15% of cases of sporadic CRC demonstrate an MSI phenotype. [30] In our study, approximately 38.9% of MSI tumors harbored the BRAF^V600E^ mutation compared with only 9.3% of MSS tumors (OR = 8.18; 95% CI = 5.08–13.17). BRAF^V600E^ mutated tumors were also more common than BRAF wild-type tumors in CIMP (OR = 16.44; 95% CI = 6.72–40.21). Therefore, the results of our meta-analysis further validated that BRAF^V600E^ mutations are correlated with CIMP and MSI. [11], [31]–[33] Furthermore, the correlation between BRAF^V600E^ status and MSI could suggest that BRAF^V600E^ mutation is a result of a deficiency in the mismatch repair (MMR) system in tumors. However, it is now clear that BRAF^V600E^ mutations rarely occur in MSI tumors with defective MMR due to a germline mutation in either the mutL homolog 1 (MLH1) or mutS homolog 2 (MSH2). [34] The frequent occurrence of BRAF^V600E^ mutation in tumors with hypermethylated MLH1 was reported previously. [35] Consistent with this, we observed a significant association between BRAF^V600E^ mutation and hypermethylated MLH1 (OR = 13.84; 95% CI = 1.75–109.24). BRAF^V600E^ and KRAS mutation were mutually exclusive in our study, consistent with previous reports. [11], [36]–[38] Nevertheless, some studies have reported tumors that harbor both BRAF^V600E^ and KRAS mutations. [19], [35], [39].

We also investigated the association between BRAF^V600E^ mutation and alcohol consumption or smoking. Although some studies reported an association between smoking history and BRAF^V600E^ mutation, [40], [41] we did not detect any significant difference in our study. Only one study included an analysis of alcohol intake, and showed a 1.87-fold increased mutation rate in non-drinkers compared with drinkers.

This meta-analysis offers several strengths. We used a comprehensive search strategy with well defined inclusion criteria, yielding the largest number of studies in such a meta-analysis to date. We used a careful approach to selecting a fixed or random effects model for pooling studies by taking into account the presence or absence of significant heterogeneity. We also performed tests for publication bias and sensitivity analysis to assess the influence of missing studies and each individual study on the pooled estimates.

Despite the strengths, there are limitations that should be considered when interpreting our results. Firstly, we did not analyze the methods used to detect BRAF^V600E^ mutations due to a lack of data, which may affect the results. Secondly, we did not collect data on the treatment and clinical outcomes to analyze effect of the BRAF^V600E^ mutation on overall clinical outcome. Finally, the relationship between BRAF^V600E^ mutation and some of the investigated parameters could not be accurately illustrated due to the limited number of studies, and so our conclusions need to be confirmed in future studies. Nevertheless, this study still reports some important and significant findings. Finally, as with all meta-analysis the validity of our pooled estimated depend on the validity of the estimates from the individuals studies, and was not possible to control for confounding in our pooled estimates.

In conclusion, this meta-analysis confirmed that the BRAF^V600E^ mutation in CRC is associated with several high-risk clinicopathological characteristics of CRC. Our data suggest that BRAF^V600E^ mutation could be used to supplement standard clinical and pathological staging for better management of individual CRC patients, and be considered as a poor prognostic marker in CRC.

## Supporting Information

Table S1 **A Summary of the 25 Studies Included in the Meta-Analysis.**
(DOC)

Checklist S1 **PRISMA 2009 Checklist.**
(DOC)
