# Supplementary material for: BRAFV600E Mutation and Its Association with Clinicopathological Features of Colorectal Cancer: A Systematic Review and Meta-Analysis
Source: PLoS One. 2014 Mar 3;9(3):e90607. doi: 10.1371/journal.pone.0090607 (PMC3940924; doi:10.1371/journal.pone.0090607)
Supplement: Table S1 — A Summary of the 25 Studies Included in the Meta-Analysis. (DOC) [file pone.0090607.s001.doc]

Table 1. A Summary of the 25 Studies Included in the Meta-Analysis

| Study | Country | Methods | Total number | | BRAF^V600E^ mutation (%) | Demographics | | Clinicopathological Features | | | | | Molecular Features | | | |
| --- | --- | --- | --- | --- | --- | --- | --- | --- | --- | --- | --- | --- | --- | --- | --- | --- |
|  |  |  |  |  |  | Gender | Age | Location | Differentiation | histological types | Clinical stage | | MSI | CIMP | MLH1 | KRAS |
| Samowitz 2005 [17] | USA | PCR+SEQ | 886 | | 9.4 | Y | N | Y | Y | Y | N | | N | N | N | Y |
| Li 2006 [34] | Australia | FSSCP+SEQ | 275 | | 8.4 | Y | N | Y | Y | Y | N | | Y | Y | Y | N |
| Kadiyska 2007 [24] | Bulgaria | PCR+SEQ | 140 | | 5.7 | Y | N | Y | N | N | N | | Y | N | N | N |
| Barault 2008 [3] | France | RT-PCR | 586 | | 13.3 | Y | N | Y | N | N | Y | | N | N | N | N |
| English 2008 [33] | Australia | RT-PCR | 582 | | 16.3 | Y | N | Y | N | N | N | | Y | N | Y | Y |
| French 2008 [9] | Not mentioned | PCR+SEQ | 490 | | 15.7 | Y | N | Y | Y | N | Y | | N | N | N | N |
| Ang 2009 [29] | Australia | FSSCP | 735 | | 6.8 | Y | N | Y | Y | Y | Y | | N | Y | N | N |
| Vilkin 2009 [6] | Israel | PCR+SEQ | 128 | | 18.8 | Y | N | Y | Y | N | N | | N | N | Y | N |
| Farina 2010 [35] | Netherlands | RT-PCR | 364 | | 15.9 | Y | Y | Y | Y | N | N | | Y | N | N | N |
| Naguib 2010 [25] | USA | PCR+SEQ | 186 | | 15.6 | Y | N | Y | Y | N | N | | Y | N | N | N |
| Roth 2010 [23] | Not mentioned | AS/RT-PCR | 1307 | | 7.9 | Y | N | Y | Y | N | Y | | N | N | N | N |
| Rozek 2010 [20] | Israel | PCR+CE | 1297 | | 5.0 | Y | N | N | N | N | N | | N | N | N | N |
| Shaukat 2010 [19] | USA | PCR+CE | 165 | | 21.8 | N | N | Y | Y | Y | Y | | Y | N | N | N |
| Zlobec 2010 [30] | Switzerland | PCR+SEQ | 374 | | 11.8 | Y | N | Y | Y | Y | N | | N | N | N | N |
| Li 2011 [36] | China | PCR+SEQ | 200 | | 7.0 | Y | Y | Y | N | N | N | | N | N | N | N |
| Tie 2011 [21] | Australia | RT-PCR+SEQ | 525 | | 9.9 | Y | N | Y | N | N | N | | Y | N | N | N |
| Yokota 2011 [26] | Japan | Cycleave PCR | 229 | | 6.6 | Y | N | Y | Y | N | N | | N | N | N | N |
| Price 2011 [22] | Australia | HRM+SEQ | 313 | | 10.5 | Y | N | Y | N | N | N | | N | N | N | N |
| Bagadi 2012 [15] | India | ARMS-PCR+SEQ | 100 | | 17.0 | Y | N | N | N | N | Y | | N | N | N | N |
| Krol 2012 [12] | Netherlands | PCR+SEQ | 126 | | 18.3 | Y | N | Y | N | N | Y | | N | N | N | N |
| Ogino 2012 [37] | Not mentioned | PCR+SEQ | | 506 | 14.8 | Y | Y | Y | N | N | | N | N | N | N | Y |
| Phipps 2012 [13] | USA | AS-PCR | | 1980 | 12.5 | Y | Y | Y | N | N | | N | N | N | N | N |
| Rako 2012 [18] | Croatia | RT-PCR | | 71 | 8.5 | Y | N | N | Y | N | | N | N | N | N | N |
| Pai 2012 [31] | USA | RT-PCR | | 181 | 11.0 | Y | N | N | N | Y | | Y | N | N | N | N |
| Bozzao 2012 [16] | Italy | HRM+SEQ | | 209 | 6.2 | Y | N | N | Y | N | | Y | N | N | N | N |
| Total number |  |  | | 11,955 | 10.8 | 24 | 4 | 20 | 13 | 6 | | 9 | 7 | 2 | 3 | 3 |

Abbreviations: BRAF, B-type raf proto-oncogene; MSI, microsatellite instability; CIMP, CpG island methylator phenotype; MLH1, mutL homolog 1; KRAS, v-Ki-ras2 Kirsten rat sarcoma viral oncogene homolog; PCR, Polymerase Chain Reaction; SEQ, sequencing; FSSCP, fluorescent-single strand conformation; RT-PCR, real-time PCR; AS, allele specific; CE, capillary electrophoresis; HRM, high-resolution melting; ARMS, amplification refractory mutation system. Y indicates that the study was evaluated for the corresponding prognostic factor; N indicates that the study was not evaluated for the corresponding prognostic factor.
